# Neuromodulation to guide circuit reorganization with regenerative therapies in upper extremity rehabilitation following cervical spinal cord injury

**DOI:** 10.3389/fresc.2023.1320211

**Published:** 2024-01-03

**Authors:** Gustavo Balbinot

**Affiliations:** ^1^ Krembil Research Institute, University Health Network, Toronto, ON, Canada; ^2^ Center for Advancing Neurotechnological Innovation to Application, University of Toronto, Toronto, ON, Canada; ^3^ KITE Research Institute – Toronto Rehabilitation Institute, University Health Network, Toronto, ON, Canada

**Keywords:** regenerative medicine, upper extremity rehabilitation, spinal cord injuries, cell therapies, stem cell therapies

## Abstract

Spinal cord injury (SCI) is a profoundly debilitating condition with no effective treatment to date. The complex response of the central nervous system (CNS) to injury and its limited regeneration capacity pose bold challenges for restoring function. Cervical SCIs are the most prevalent and regaining hand function is a top priority for individuals living with cervical SCI. A promising avenue for addressing this challenge arises from the emerging field of regenerative rehabilitation, which combines regenerative biology with physical medicine approaches. The hypothesis for optimizing gains in upper extremity function centers on the integration of targeted neurorehabilitation with novel cell- and stem cell-based therapies. However, the precise roles and synergistic effects of these components remain poorly understood, given the intricate nature of SCI and the diversity of regenerative approaches. This perspective article sheds light on the current state of regenerative rehabilitation for cervical SCI. Notably, preclinical research has yet to fully incorporate rehabilitation protocols that mimic current clinical practices, which often rely on neuromodulation strategies to activate spared circuits below the injury level. Therefore, it becomes imperative to comprehensively investigate the combined effects of neuromodulation and regenerative medicine strategies in animal models before translating these therapies to individuals with SCI. In cases of severe upper extremity paralysis, the advent of neuromodulation strategies, such as corticospinal tract (CST) and spinal cord stimulation, holds promise as the next frontier in enhancing the effectiveness of cell- and stem cell-based therapies. Future preclinical studies should explore this convergence of neuromodulation and regenerative approaches to unlock new possibilities for upper extremity treatment after SCI.

## Introduction to regenerative rehabilitation for upper extremity recovery using regenerative approaches

### Regenerative rehabilitation for upper extremity recovery

Regenerative rehabilitation is an approach that combines regenerative medicine with rehabilitation strategies to promote functional recovery of impaired individuals. Spinal cord injuries (SCIs) have drastic impacts on sensorimotor function and are most commonly damaging the cervical spinal cord. Individuals living with cervical SCIs face the challenge of limited recovery in hand strength, a function heavily reliant on spared corticospinal tract (CST) projections (1). Although regaining hand function is a top priority for individuals living with cervical SCIs (2), most of the preclinical studies using regenerative rehabilitation approaches focused on the lower extremities using thoracic SCI models [Reviewed in (3, 4)].

The intricacy of dexterous hand movements underscores the critical nature of restoring any remaining CST projections to motoneurons governing hand muscles in the context of SCI. This is fueled by the translational challenges of rodent models of upper extremity dysfunction after SCI—which may contribute to the scenario described above (5). Given the central nervous system's (CNS) restricted capacity for regenerating these essential connections, novel cell and stem cell-based therapies have emerged as potential solutions to promote plasticity and replace damaged cells (6).

Anticipation surrounds the role of these cell- and stem cell-based therapies in regaining lost function, bolstered by the promising safety and efficacy outcomes observed in recent clinical trials (3, 6). These advances offer hope for individuals living with the limitations of cervical SCIs, potentially paving the way for more effective treatments and meaningful improvements in hand strength and function.

### Neuromodulation

I will focus this perspective article on current methods used in neurorehabilitation in the clinic. Current practices are evolving to emphasize innovative methods for providing neurorehabilitation to individuals living with SCIs. This paradigm shift is especially prominent in cases of severe paralysis, where rehabilitation strategies are increasingly harnessing the power of neuromodulation to intensify the recovery process. Upper extremity rehabilitation, in particular, involves a multifaceted approach that combines sensory feedback from the periphery with upper motor neuron commands to facilitate volitional upper limb movement. In situations of severe paralysis, where volitional actions may be weak or absent, electrical stimulation plays a pivotal role in delivering the necessary external stimulus to promote recovery. This holds particular significance for hand function, as it depends on robust afferent feedback to the spinal cord (7) and is significantly reliant on lateral tract efference (1).

Neuromodulation systems have emerged as a game-changing tool, capable of delivering functional electrical stimulation (FES) to upper extremity muscles with precise timing, evoking reaching and grasping function (8). Similarly, spinal cord stimulation has proven effective in promoting targeted circuit reorganization in the lumbar spinal cord, culminating in the restoration of standing and walking capabilities, even in the absence of direct stimulation (9, 10). However, the inherent complexity of the brain and cervical circuits responsible for controlling dexterous hand movements implies that achieving upper extremity recovery through these approaches alone may be more challenging.

A noteworthy development is an ongoing clinical trial investigating the utility of epidural cervical spinal cord stimulation [UP2—Brain Controlled Spinal Cord Stimulation in Participants With Cervical Spinal Cord Injury for Upper Limb Rehabilitation—NCT05665998]. The integration of these neuromodulation strategies with cell- and stem cell-based therapies is poised to mark the next phase in the field of regenerative medicine for upper extremity recovery following SCIs. This combined approach holds the potential to optimize functional gains for individuals living with SCI, fostering hope for meaningful improvements in upper extremity function and quality of life.

### Understanding cervical spinal cord injuries

The most striking part of human evolution involved the development of dextrous hand use with a respective expansion of the sensorimotor cortex controlling hand movements, which, because of the extensive CST projections, may constitute a drawback after cervical SCI. Understanding SCIs is crucial in comprehending the importance of regenerative rehabilitation approaches for upper limb function. Spinal cord injuries refer to the damage or trauma caused to the spinal cord resulting in varying degrees of sensory and motor function loss. The severity and extent of the injury depend on the location and severity of the damage to the spinal cord, with cervical SCIs displaying more severe impairment both in the lower and upper extremities.

The segmental recovery after cervical SCIs (C1-T1) displays a proximal-distal gradient, where myotomes innervating proximal upper limb muscles, such as the deltoid and biceps brachii muscles, displays superior recovery compared to distal hand muscles (1, 8). Thereby, given its severity and priority for recovery, the optimization of distal hand muscles recovery will likely need targeted and combinatorial approaches. Below, I will briefly explore current treatment options for upper extremity recovery after cervical SCIs and offer my perspective on why the regenerative rehabilitation approach may be explored as a promising option to create a biological bridge enabling greater recovery of upper extremity function.

### Current treatment options for cervical spinal cord injuries

Current treatment strategies for cervical SCIs come with limitations, often resulting in partial recovery, particularly in the case of distal hand muscles. In the chronic stages, approaches such as nerve transfers or tenodesis surgery are explored to create peripheral nerve or tendon bridges, with the goal of enhancing lost movements in the distal extremities (11, 12).

Here, we advocate for the imperative need for regenerative rehabilitation therapies to circumvent the limitations of current treatment options. Urgently addressing the promotion of neurite regeneration and functional recovery following SCI, especially to facilitate enhanced upper extremity recovery during the chronic phase.

The concept of creating a biological bridge to transmit critical CST projections to lower motor neurons responsible for innervating distal hand muscles holds the potential to re-establish manual dexterity and significantly enhance the functional independence of individuals living with cervical SCIs.

## Exploring regenerative rehabilitation for cervical spinal cord injuries

Regeneration, the process of generating again, holds profound significance in the context of SCI. However, the CNS, with its intricate structure primarily established during development, exhibits a remarkably limited capacity for regeneration. These inherent constraints serve as the brakes on plasticity, safeguarding the critical structure and function of the fully developed nervous system.

After an SCI, there is a fascinating phenomenon of natural recovery in the upper extremity, more pronounced during the initial six months post-injury and continuing for over a year (1). This recovery signals that the same lesion responsible for damaging cells also triggers a neuroplasticity process, leading to the partial re-establishment of lost connections. Some cells may even undergo a transformation, adopting an embryonic signature as part of the regenerative response after the injury, as indicated by (13).

However, this natural recovery process is not endless, and individuals ultimately find themselves with persistent upper extremity impairments. Despite the glimpses of plasticity and regeneration, the CNS's intrinsic limitations underscore the need for innovative approaches to augment and prolong the regenerative processes following an SCI.

Cell-based, stem cell-based and pharmacological therapies attempt to re-open this window of plasticity by deactivating growth-inhibiting factors, introducing new pluripotent stem cells, or promoting a favorable environment for plasticity. The use of biomaterials may support stem cell integration and help by releasing growth factors or enzymes (6, 14). Nonetheless, these processes must be guided by the appropriate sensorimotor inputs to avoid aberrant plasticity and lead to functional outcomes—regenerative rehabilitation.

**Figure 1:**
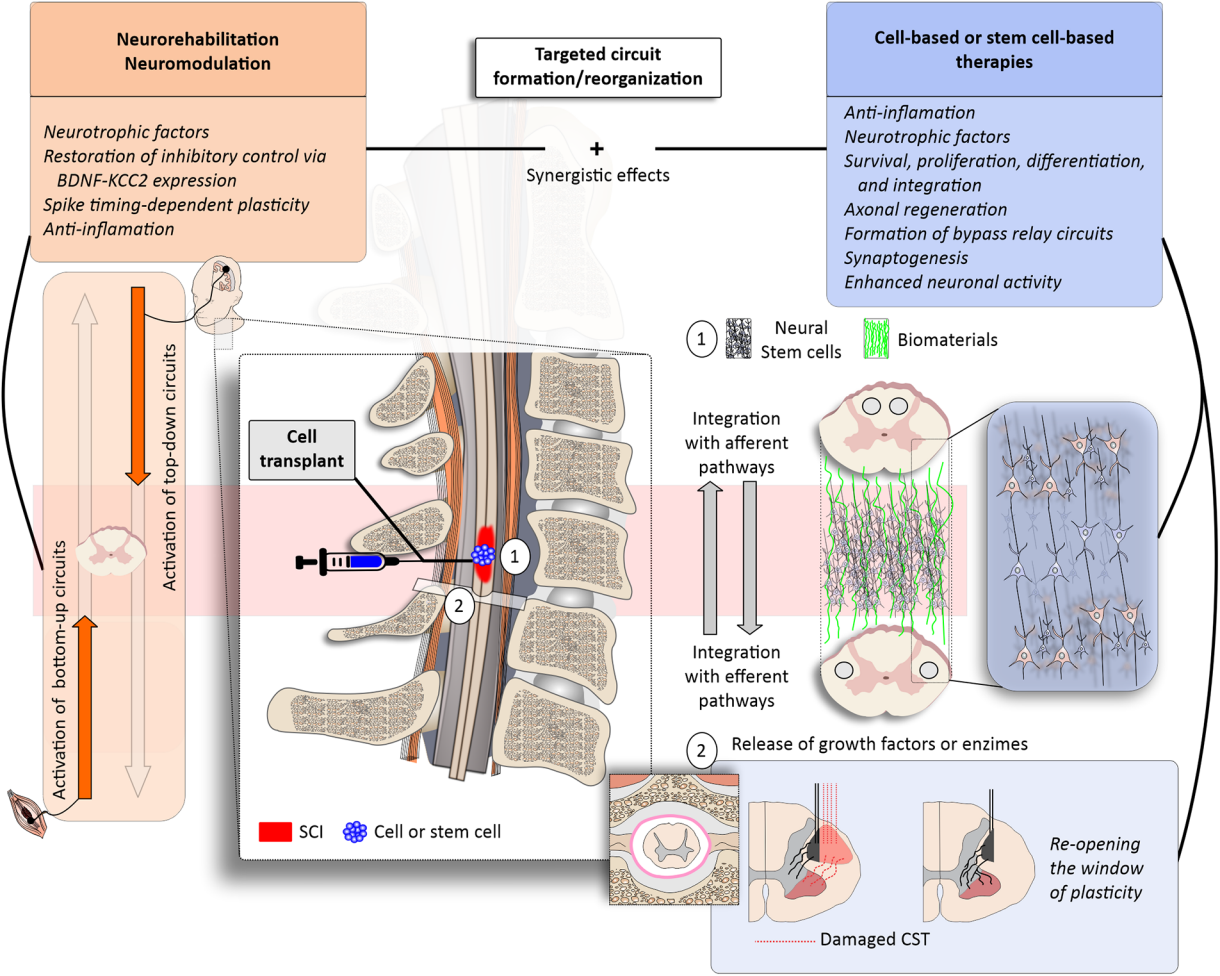
Targeted circuit formation/reorganization with regenerative rehabilitation. (left panels) Neurorehabilitation may be boosted by the use of neuromodulation approaches, such as electrical or magnetic stimulation to activate specific bottom-up and top-down circuits (orange). Physical rehabilitation and exercise have been associated with anti-inflammatory effects and the release of neurotrophic factors, such as the brain-derived neurotrophic factor (BDNF)—which may promote plasticity and the re-establishment of excitatory/inhibitory balance through the upregulation of the potassium-chloride cotransport isoform 2 (KCC2). Functional electrical stimulation (FES) therapy and paired associative stimulation protocols activate these up-and-down pathways in physiological timing, creating the opportunity for spike timing-dependent plasticity. (right panels) The combination of neurorehabilitation and neuromodulation with cell-based and stem cell-based therapies may promote synergistic effects to enhance, for example, cell survival, proliferation, differentiation and integration (blue). (1) Stem cell transplants may be combined with biomaterials (green); neurorehabilitation and neuromodulation may guide cellular survival, proliferation, differentiation and integration. (2) Pharmacological therapies may be injected intrathecally to remove the brakes of plasticity by acting on extra-cellular matrix proteins (e.g., Chrondoitinase ABC) or enhance axonal regeneration (e.g., anti-NOGO). The combined approaches would favour targeted circuit formation/reorganization after SCI.

Previous reviews have shown that regenerative rehabilitation may act via several pathways, including the release of brain-derived neurotrophic factor and growth-associated protein 43, reduction of calcitonin gene-related peptides, greater differentiation of precursor cells into neurons and oligodendrocytes, increased serotonergic activity, among others [Reviewed in (3, 4)]. Here, instead of exploring these mechanistic effects in detail, I will instigate further research on the role of rehabilitation & neuromodulation in promoting targeted circuit formation (Figure 1).

### Regenerative rehabilitation using pharmacological agents, antibodies or enzymes

Regenerative rehabilitation, employing various pharmacological agents, antibodies, and enzymes, offers new hope for the treatment of SCIs. Despite the pressing need for effective interventions, current pharmacological treatments have fallen short in enhancing neurological repair in acute SCI, as reported in studies by (6, 15). Among these treatments, RhoA inhibitors have shown promise in promoting axonal growth after SCI, yielding positive results in animal models by reducing syrinx-cavity formation and preserving white matter (16). However, clinical trials faced challenges in demonstrating primary efficacy endpoints (17). Similar hurdles emerged with Riluzole, a sodium-glutamate antagonist with neuroprotective potential (18).

To unlock the full potential of pharmacological treatment, improvements in local drug delivery, clinical trial design, and the integration of targeted rehabilitation may be pivotal in optimizing the outcomes of these interventions for cervical SCI. Nogo-A, an inhibitor of neurite growth and plasticity in the adult CNS, presents a significant brake to regeneration. Nogo-A is an oligodendrocyte membrane protein that interacts with neuronal receptors, one of the best-known inhibitors of neurite growth and plasticity in the adult CNS—by restricting long-distance axon growth and regeneration to stabilize neuronal circuits (19, 20). Treatment with anti-Nogo-A antibodies has shown promise in neutralizing these inhibitory effects, leading to collateral sprouting, functional recovery, and well-tolerated outcomes in humans (21). The results of an ongoing clinical trial (NISCI—Nogo Inhibition in Spinal Cord Injury—NCT03935321) hold great promise for the community.

Additionally, the extracellular matrix, particularly chondroitin sulfate proteoglycans, impedes axonal regeneration and plasticity, inhibiting the endogenous repair of the injured spinal cord. Chondroitinase ABC, an enzyme that can dissolve chondroitin sulfate proteoglycans, presents promise. In conjunction with growth factors, it has displayed benefits in rat models of SCI, with the potential for even greater effects when combined with stem cell therapies or targeted rehabilitation, promising sustained functional improvements (22).

### Regenerative rehabilitation with cell-based and stem-cell-based therapies

Cell-based therapies hold immense promise for SCI treatment. These therapies encompass the transplantation of mature cells and stem cell-based interventions, which involve undifferentiated or partly differentiated cells capable of differentiation and proliferation. Emerging as one of the most promising strategies for SCI treatment, these approaches aim to repair the injured spinal cord (6).

Current evidence underscores the primary role of transplanted stem cells in differentiating into oligodendrocytes to promote remyelination, as demonstrated by (23–25). Recent breakthroughs have illuminated the potential for neural progenitor cell transplantation in combination with rehabilitation, as it was found to foster host corticospinal axon regeneration into grafts. Even in severe cervical contusion models, this approach yielded meaningful forelimb sensorimotor recovery, an outcome of paramount clinical significance (26). Thereby, the integration of targeted and intense rehabilitation strategies into clinical trial designs offers promise in augmenting the modest effects seen in prior clinical trials (6).

However, severe paralysis commonly results in a lack of volitional control, making rehabilitation complex and requiring the implementation of neuromodulation strategies. For instance, FES therapy, as demonstrated in studies like (8), enables the functional activation of upper extremity muscles, even in the most severe paralysis cases, guiding circuit reorganization. Additionally, electrical spinal cord stimulation has proven to have enduring effects, persisting beyond the cessation of stimulation (9, 10, 27). Future research endeavors should explore the combined potential of neuromodulation & rehabilitation in conjunction with stem cell transplantation.

The envisioned synergistic effects of regenerative rehabilitation are depicted in Figure 1. Rehabilitation serves as an external stimulus for targeted circuit reorganization, activating both top-down and bottom-up circuits. This stimulation instigates use-dependent plasticity in the spinal cord, such as spike-timing-dependent plasticity, and orchestrates the release of neurotrophic factors, including brain-derived neurotrophic factor, among others. These growth promoting factors play a pivotal role in promoting and enhancing plasticity and re-establishing the inhibitory-excitatory balance by upregulating the potassium-chloride cotransport isoform 2 (KCC2).

Cell- and stem cell-based therapies may also foster an anti-inflammatory environment, potentially guiding axonal regeneration and synaptogenesis. The cumulative effect is heightened neuronal activity and the formation of bypass circuits, ultimately culminating in enhanced functional recovery. To further enhance clinical trials in SCI, the incorporation of biomaterial scaffolds is a crucial consideration. Biomaterials have the potential to provide sustained support for transplanted stem cells, release growth factors, and enzymatically remove extracellular matrix components that limit plasticity (6).

## Case studies: regenerative rehabilitation in current clinical practice

### Unlocking enhanced upper extremity recovery through regenerative rehabilitation

Timing is everything in the pursuit of recovery after an SCI. An upper extremity rehabilitation regimen administered within the critical window of neural plasticity during the natural recovery process has the power to propel individuals toward enhanced recuperation. But there's more to this story. When regenerative therapies, including cell and stem cell treatments, are coupled with targeted rehabilitation, the possibilities for functional recovery expand dramatically, even to the chronic phases of the injury.

Recent preclinical research, such as the study by (26), has ignited excitement within the field. In a cervical SCI model, animals receiving combined treatment—targeted upper extremity rehabilitation and stem cell transplants—outperformed their counterparts who received stem cells alone. These findings hold the promise of significant advancements in upper extremity function recovery.

However, this is only the beginning. Translating these results into forthcoming clinical trials is the next vital step, raising hopes for transformative breakthroughs. Yet, the journey towards a deeper understanding of the optimal timing, intensity, and histological outcomes of this combined therapy in animal models must continue.

**Figure 2:**
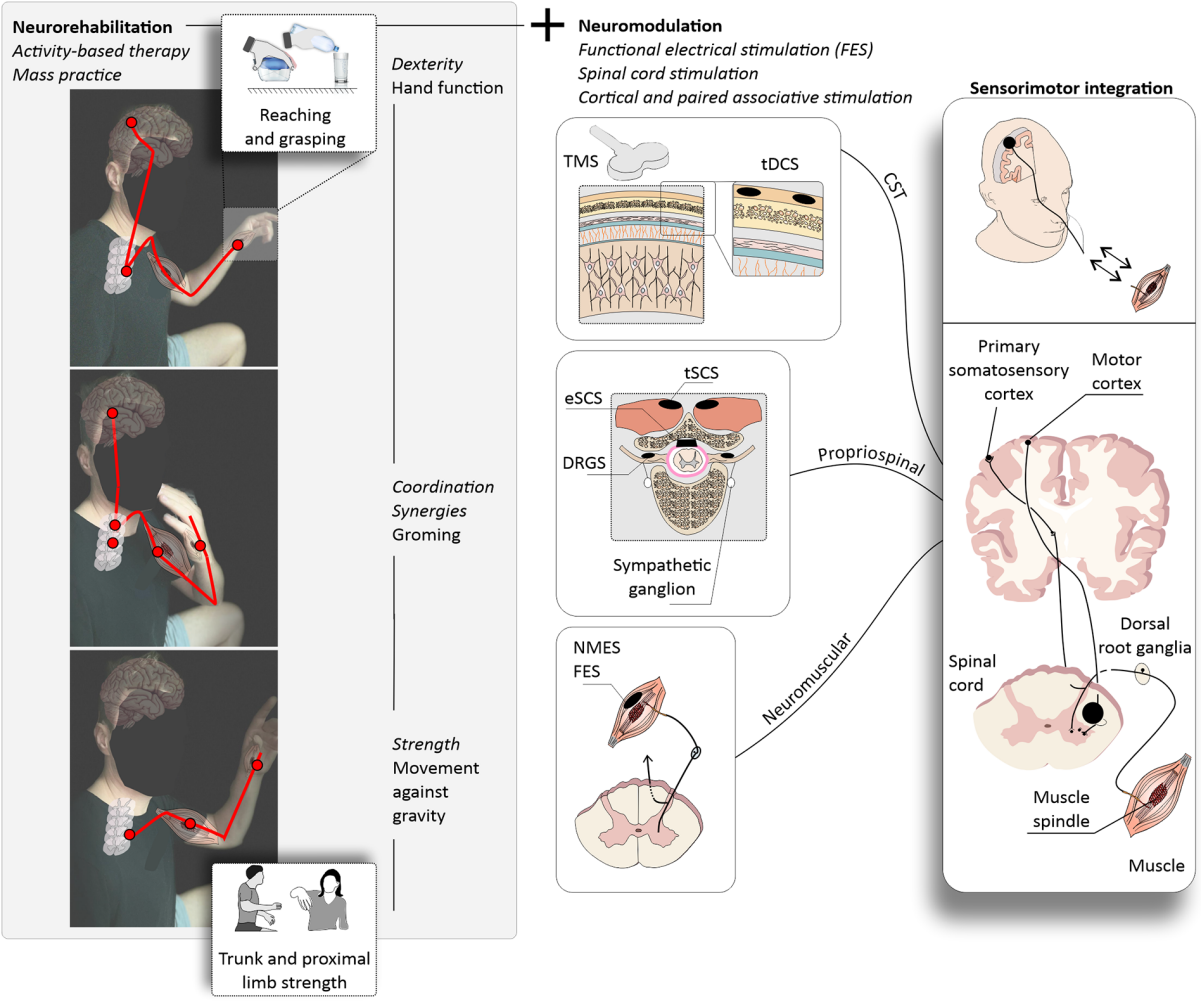
Upper extremity neurorehabilitation following cervical SCI. (left panels) Activity-based therapy for upper extremity recovery may consist of repetitive training of functional movements through mass practice, for example reaching and grasping. Therapist- or robotic-assisted movements are repeated over many rehabilitation sessions with the ultimate goal of improving the functionality of the upper extremity. A typical neurorehabilitation protocol may target the performance of movements against gravity using the proximal upper extremity joints (bottom) and progress to functional grooming, and ultimately reaching and grasping (middle and upper panels). Each movement requires the use of specific but overall intertwined central and peripheral nervous system structures (red lines). (right panels) Targeted rehabilitation aims at the recovery of upper extremity strength and hand dexterity and is often combined with neuromodulation techniques to enhance function. Cervical SCIs may have drastic impacts on upper extremity function depending on the severity. For severe upper extremity paralysis, the use of neuromodulation strategies is common and may involve the use of functional electrical stimulation (FES), spinal cord stimulation (SCS), and cortical or paired associative stimulation. Corticospinal tract (CST) stimulation may enhance corticospinal excitability via transcranial magnetic stimulation (TMS) or transcranial direct current stimulation (tDCS) and may be paired with peripheral stimulation to promote the strengthening of CST connections (paired associative stimulation). These neurorehabilitation and neuromodulation strategies target specific motor and sensory pathways providing the stimulus for sensorimotor integration with the ultimate goal of enhancing upper extremity function. TMS, transcranial magnetic stimulation; tDCS, transcutaneous direct current stimulation; eSCS, epidural spinal cord stimulation; DRGS, dorsal root ganglia stimulation; NMES, neuromuscular electrical stimulation; FES, functional electrical stimulation; CST, corticospinal tract.

Another avenue of great promise lies in electrical neuromodulation. Researchers such as (28), have discussed the potential for enhancing rehabilitation and functional recovery through neuromodulation techniques. These advancements are paving the way for a new era in SCI treatment. To complement these insights, we provide a concise overview of the case modalities of targeted rehabilitation and neuromodulation, common staples of current clinical practice in SCI rehabilitation (refer to Figure 2).

### Activity-based therapy: trunk and proximal upper extremity stability, reaching and grasping, and hand dexterity

Mass practice! Repetitions! Tailored to weaker upper extremity muscles to regain specific functions—termed activity-based therapy. This is upper extremity rehabilitation in its cruder state, with the help of a therapist or rehabilitation robots. Activity-based therapy, in its essence, represents a return to basics, where the emphasis is on sheer repetition and tailored exercises. The goal is clear: relearning the intricate art of upper extremity movements. Relearning to perform upper extremity movements is a challenging task and may involve the regain of trunk stability, movements against gravity using the arm and shoulder and progress to reaching and grasping. To comprehend the significance of this approach, we must first recognize that the recovery path following an SCI is marked by distinct phases. During the initial six months post-injury, individuals often experience significant improvements in upper extremity function. However, this progress tends to level off, leading to a more modest recovery profile between six and eighteen months. Nonetheless, one critical aspect continues to elude many—hand dexterity. For those living with cervical SCIs, the quest to regain hand function remains a paramount priority (2). Regenerative medicine approaches are useful for both boosting the effects of activity-based therapy in the sub-acute phase after SCI and promoting additional recovery after the natural recovery plateaus.

### Functional electrical stimulation (FES) therapy

In severe paralysis, volitional upper extremity movement may be absent or very weak. Therefore, targeted upper extremity rehabilitation may involve the use FES therapy, which supplements the weak or absent upper motor neuron control with dosed and timed activation of muscle fibers and sensory afferents. This efferent volley promotes the contraction of the upper extremity muscles at the appropriate timing for performing the given function. The strong ascending afferent volley conducted by large-diameter sensory fiber signals to the spinal cord ascending systems and reinforce connections related to sensorimotor integration (8). The activation of these top-down and bottom-up systems by FES therapy in combination with cell and stem cell therapies may help the establishment of the propriospinal circuitry and spinal cord pathways– leading to enhanced recovery of the specific upper extremity function.

### Spinal cord stimulation

Targeted circuit reorganization is also promoted by electrical stimulation of the spinal cord, with long-lasting effects even with the cessation of the stimulation (9). Nonetheless, most of the studies supporting these findings were conducted in the lumbar enlargement with the target of restoring standing and walking. The upper extremities, particularly in the context of dexterous hand movements, represent a distinct challenge. Contrary to the lower extremities, which receive more support from specific propriospinal neurons, the upper extremities heavily depend on the intricate projections of upper motor neurons. This unique reliance on upper motor neurons makes the restoration of upper extremity function a complex puzzle. Studies using cervical spinal cord stimulation are undergoing and more evidence is needed to support its use for upper extremity function. Because upper extremity function, especially dexterous hand movement, is supported and heavily relies on upper motor neuron projections, it is unknown how the activation of large diameter sensory axons by spinal cord stimulation can enhance reaching and grasping function. Perhaps by providing trunk and proximal upper extremity stabilization to support dexterous hand movement or even a strong effect on sensorimotor integration, facilitating the gating of the much-needed sensory information to the spinal cord circuits controlling hand movement. More studies are necessary, from a neurophysiological point of view it seems that, at least for regaining hand function, the concomitant stimulation of the motor cortex will be an important step in achieving superior hand functional recovery by strengthening the residual CST projections. While these theories hold promise, the path to achieving superior hand functional recovery is not without its challenges. The need for further exploration is evident, particularly from a neurophysiological standpoint.

### Unleashing the potential of upper motor neurons: a path to enhanced recovery

Upper motor neurons controlling the hand muscles are abundant in the motor cortex (29, 30). Less so are the motor units responsible for transmitting the motor information to hand muscles [non-human primates (31)]. Each hand muscle is innervated by only a few hundred motor units, and even so, is capable of producing movements in many degrees of freedom with astonishing precision. Indeed, the integrity and density of residual CST projections to hand muscles are good predictors of hand strength recovery (1). In individuals with the preservation of residual CST projections to hand muscles, the combination with cell therapies to promote the re-opening of the window of plasticity by stopping anti-growth signaling may constitute an efficient type of rehabilitation. For more severe lesions to the CST, stem cells may help to bypass the damaged circuits and extend many centimeters along the craniocaudal axis of the spinal cord to promote the partial re-establishment of CST projections controlling hand muscles to allow enhanced recovery of hand dexterity. These approaches are to be combined with intense rehabilitation and cortical or timed paired associative stimulation (32) to promote targeted circuit reorganization- similar to walking (33). In doing so, they facilitate the partial re-establishment of CST projections that govern hand muscles, ultimately enhancing the recovery of hand dexterity.

Findings from animal studies indicate that cortical stimulation in SCI rats improve recovery in forelimb function, which was linked to increased CST plasticity evidenced by a signiﬁcant increase in the sprouting of collaterals above the lesion site, but not to increased regenerative growth through the lesion itself (34). In this line of thought, the integration of stem cell transplants into the equation opens up new vistas of opportunity. These transplants may bridge the divide created by the lesion, paving the way for axonal linkage and growth. This biological bridge extends CST information from above the lesion site to intact lower motor neurons below the level of injury, re-wiring the path toward the target cells.

## Discussion

### Navigating the hurdles: challenges and limitations of regenerative rehabilitation

The costs of rehabilitation, particularly in the context of SCIs, are indeed a significant concern for healthcare systems. Comprehensive rehabilitation programs for individuals with SCIs encompass a wide spectrum of elements, including medical care, physical therapy, occupational therapy, assistive technologies, and often long-term care and support. The financial burden can be overwhelming, as these programs necessitate specialized facilities, state-of-the-art equipment, and a dedicated team of highly trained healthcare professionals.

Scientific evidence plays a pivotal role in advocating for the inclusion of rehabilitation in upcoming clinical trials for SCIs. Rigorous scientific studies can provide concrete evidence of the efficacy of rehabilitation in improving the functional outcomes in preclinical models. This evidence is essential to convince authorities and stakeholders of the value of rehabilitation in the recovery process. Quantifiable data from clinical trials can demonstrate how rehabilitation positively impacts the quality of life of individuals with SCIs.

In the broader context, these quantifiable outcomes can play a pivotal role in helping policymakers and healthcare authorities appreciate that rehabilitation is not merely an expenditure but an investment in the well-being of those affected. Clinical studies must meticulously track the dosage of rehabilitation provided and should include control groups not receiving rehabilitation, a design often better suited to preclinical studies due to ethical concerns related to withholding rehabilitation from a group.

By reporting persuasive scientific evidence, the regenerative rehabilitation community can underscore the critical role of rehabilitation as an indispensable component of a comprehensive, evidence-based approach to managing SCIs. Policymakers and authorities frequently rely on such scientific data to make informed decisions regarding resource allocation, reimbursement policies, and the inclusion of specific treatments or interventions within healthcare systems.

To secure the inclusion of rehabilitation in forthcoming clinical trials for SCIs, it is imperative for the scientific community, healthcare providers, and patient advocacy groups to collaborate closely. The design and execution of well-structured studies that yield compelling evidence can serve as a potent tool for convincing authorities of the pivotal role of rehabilitation in the treatment and recovery process for individuals facing SCIs.

This evidence can then form the bedrock upon which healthcare policies and funding priorities are constructed, ensuring that rehabilitation remains accessible to those who require it. By navigating these challenges with determination and unity, the field of regenerative rehabilitation can pave the way for a brighter and more inclusive future for individuals affected by SCIs.

### The future of regenerative rehabilitation in spinal cord injury management

When the SCI is incomplete, electrical stimulation is channeled into muscles, nerves, spinal cords, brains, or brainstems to reignite circuits in the periphery and CNS, bolstering the connections that traverse the intricate web from the brain to the spinal cord to the muscles. When an SCI is severe, the circuits in the spinal cord lack the source of modulation and excitation that they require to be functional. Electrical spinal cord stimulation stands as a state-of-the-art technique in this endeavor. By modulating large-diameter afferents, it can rekindle these dormant circuits. When coupled with dedicated neurorehabilitation, this stimulation charts a course for the resurgence of residual projections emanating from the brain and brainstem. For example, recently it was show that these projections converge with SCVsx2::Hoxa10 neurons, awakened by the stimulation (35). This intricate dance of reorganization weaves the tapestry of recovery, breathing life into volitional movements, even when the stimulation is in turned off. The transplantation of cells holds the promise of a parallel journey. With the right guidance from intense, targeted rehabilitation, these cells may assume a parallel fate, perhaps acting as proxies of recovery for upper extremity function after cervical SCIs.

Despite the notable absence of an effective pharmacological treatment for SCIs, cell- and stem cell-based therapies show promise. These therapies hold the potential to supplant lost cells and restore upper extremity function following paralysis. We know that neural stem cells have the remarkable ability to integrate into host tissue and extend axons deep into the host spinal cord, transcending considerable distances (e.g., C5–C8) (24, 26, 36, 37). Yet, for these cells to truly fulfill their potential, they crave the right synaptic inputs to create functional harmony within the spinal cord (33). Herein, lies the importance of targeted rehabilitation. Here, I support the idea that targeted rehabilitation provides the external stimulus needed for this process to occur in the spinal cord– without resorting of chemoattracting approaches (33). While this intricate process remains poorly understood, it may serve as a guiding light in the formation of functional circuits within the spinal cord. Circuits that, in time, may breathe life into the promise of restoring upper extremity function—especially the much-needed hand function.

### Conclusion: revolutionizing SCI management—the promise of regenerative rehabilitation

Regenerative rehabilitation presents a promising avenue for individuals living with cervical SCIs. By combining regenerative medicine with targeted rehabilitation strategies, this innovative approach holds the potential to significantly enhance the recovery of upper extremity function.

While traditional treatment methods have shown limitations, regenerative rehabilitation offers a new therapeutic venue. It recognizes the intrinsic regenerative capacity of spinal cord neurons, aiming to create an environment that stimulates growth and repair in the damaged cord. This approach goes beyond symptom management and focuses on addressing the root causes of the injury, promoting neurite regeneration, and reestablishing critical connections within the spinal cord.

The synergy of regenerative rehabilitation with various therapeutic approaches, including pharmacological interventions, and neuromodulation, provides a comprehensive strategy to optimize recovery.

One of the key elements in the success of regenerative rehabilitation is the provision of an external stimulus through rehabilitation practices and neuromodulation strategies. These stimuli shape plasticity, propelling use-dependent plasticity within the spinal cord, and setting the stage for the release of neurotrophic factors like brain-derived neurotrophic factor. These factors promote, enhance, and restore the balance between inhibition and excitation, awakening the dormant axonal regeneration and synaptogenesis. Regenerative rehabilitation is not a solo act, but a symphony, harmonizing the finest facets of multiple fields to achieve the most promising results.

Yet, it's crucial to remain aware of the challenges and boundaries that regenerative rehabilitation deals with, including its financial costs and the ongoing need for refining and adapting the approach across various injury types and stages.

The future of regenerative rehabilitation in the management of SCIs is filled with promise. As our comprehension of the intricate interplay between regenerative medicine, rehabilitation, and neuromodulation deepens, we anticipate increasingly effective, personalized treatments. In summary, regenerative rehabilitation charts a transformative course in the SCI landscape. By uniting state-of-the-art regenerative therapies with precision-targeted rehabilitation practices and neuromodulation, it opens a gateway to unprecedented advancements in upper extremity function and overall quality of life for those living with cervical SCIs. As research advances, regenerative rehabilitation emerges as a promising field of research, promising to restore what was once believed to be irrevocably lost.

## Data Availability

The original contributions presented in the study are included in the article/Supplementary Material, further inquiries can be directed to the corresponding author.
